# Integrating the IoT and Blockchain Technology for the Next Generation of Mining Inspection Systems

**DOI:** 10.3390/s22030899

**Published:** 2022-01-25

**Authors:** Miguel Pincheira, Mattia Antonini, Massimo Vecchio

**Affiliations:** Fondazione Bruno Kessler, 38123 Trento, Italy; mpincheiracaro@fbk.eu (M.P.); m.antonini@fbk.eu (M.A.)

**Keywords:** Mining 4.0, digitization, distributed ledger technologies, smart contract, IoT retrofit

## Abstract

Inspection of mining assets is a crucial part of the maintenance process and is of interest to several stakeholders (e.g., OEMs, owners, users, and inspectors). Inspections require an inspector to verify several characteristics of the assets onsite, typically using legacy and poorly digitized procedures. Thus, many research opportunities arise from the adoption of digital technologies to make these procedures more efficient, reliable, and straightforward. In addition to cloud computing, the ubiquitous presence of modern mobile devices, new measurement tools with embedded connectivity capabilities, and blockchain technologies could greatly improve trust and transparency between the stakeholders interested in the inspection. However, there has been little discussion on integrating these technologies into the mining domain. This paper presents and evaluates an end-to-end system to conduct inspections using mobile devices that directly interact with constrained IoT sensor devices. Furthermore, our proposal provides a method to integrate constrained IoT devices as smart measuring tools that directly interact with a blockchain system, guaranteeing data integrity and increasing the trustworthiness of the data. Finally, we highlight the benefits of our proposed architecture by evaluating a real case study in a mining inspection scenario.

## 1. Introduction

The mining sector is one of the most challenging domains to develop next-generation applications using new technologies such as the Internet of Things (IoT). Dust, noise, underground operations, high humidity, temperature, isolated locations, lack of connectivity, and extreme meteorological conditions are just a few challenges that make the adoption hard and require additional research [1]. One fundamental aspect in the mining domain is the maintenance of assets (i.e., facilities, machines, and equipment), as they directly impact mines’ operational activities and workplace safety. According to a recent analysis of TheWorldCounts (https://www.theworldcounts.com/challenges/planet-earth/mining/health-effects-of-mining/story, accessed on 14 December 2021), more people are injured or killed in the mining industry than in any other sector, with over 15,000 miners dying every year. Furthermore, poorly maintained machines may stop working during operations, causing unhealthy consequences for the surrounding environment. On average, the mining and quarrying sectors experience a loss of hundreds of million euros per year due to accidents and injuries, turning into a huge social and economic impact on mining companies, according to a recent AVEVA’s report (https://www.aveva.com/en/industries/infrastructure/, accessed on 14 December 2021).

Proper maintenance processes are one of the key elements to achieve the conditions required to guarantee the safety of workers and the protection of the environment. As defined by European Standards (https://www.en-standard.eu/din-31051-fundamentals-of-maintenance/, accessed on 14 December 2021), maintenance operations are composed of four tasks: service, inspection, overhaul, and improvement. Maintenance operations are planned based on periodic inspections, adopting two main approaches:

- Following the original equipment manufacturer (OEM) guidelines and recommendations to properly maintain equipment. OEM’s manuals and operators’ experience typically support this process and aim to keep the assets in working conditions, reducing maintenance costs.
- Following the outcomes of inspections carried out by a designated inspection officer appointed by a local, national, or supranational body. Public agencies mainly support these inspections with a duty to guarantee certain levels of safety of mining sites [2] and efficiency of machines (e.g., exhaust gases, tire pressures, minimum tire tread height).

Therefore, the inspection of mining assets is a process that involves several stakeholders, including the OEM, the owner, the user, inspectors, and various services companies [3]. In mining, and regardless of the followed approach, inspections are usually carried out using legacy and poorly digitized procedures. First, an inspector receives the information about the asset, printing the checklist of inspection points to collect. Then, she goes to the mining site to inspect the asset, noting down all the critical inspection points. Next, the inspector uses certified measurement tools (e.g., calipers, noise dosimeters, depth gauges) or writes observations that cannot be measured (e.g., missing instructions book, broken taillights). Once the inspection is complete, the collected measures and observations are manually inserted into an IT system, responsible for storing the detailed inspection data, computing the inspection’s outcome, and continuing the maintenance process.

In this context, many research opportunities arise from the adoption of digital technologies to make inspection procedures more automated, reliable, and simple. First, the ubiquitous presence of modern mobile devices (e.g., smartphones, tablets) provides a low-cost tool to carry out inspections, store measures and observations, and then automatically transfer them to an IT system to obtain the inspection result. This approach can work even in remote mining sites with no Internet connection because mobile devices have enough memory to collect several inspections and transfer them once an Internet connection is available. Using these technologies could greatly simplify the process, reducing the overhead of writing the information several times. Second, modern mobile devices offer various short-range communication technologies (e.g., Bluetooth, WiFi, NFC) to interact with the new measurement tools (e.g., calipers and depth gauges). This approach can also work with older tools as cost-effective IoT devices could be used for retrofitting these tools [4,5]. Using these technologies could greatly improve the precision of the measurements and reduce the errors due to human intervention. Third, blockchain technologies provide a trusted repository of information, where data are secure and traceable, and the data source can be precisely identified [6]. Using blockchain technology could further improve trust and transparency between the stakeholders involved in the maintenance process [3].

Despite this interest, there has been little discussion on integrating these technologies into the mining domain. This paper presents and evaluates an end-to-end system to conduct inspections using mobile devices that directly interact with connected measurement tools. Furthermore, our proposal provides a method to use low-cost constrained IoT devices to enable measuring tools to directly interact with a blockchain system, guaranteeing data integrity and increasing the trustworthiness of the measurement. Finally, we highlight the benefits of our proposed architecture by evaluating a real case study in a mining inspection scenario. This pilot represents one of the outcomes of an ongoing research and innovation project funded by the EU Commission and involving research institutions, universities, and relevant companies in the mining sector. Consequently, the main contribution of this paper is two-fold:

1. We propose a system architecture for mining machines inspections using off-the-shelf mobile devices and integrating IoT and blockchain technologies.
2. We propose a method to create cost-effective IoT measurement devices using low-cost embedded CPUs that interact with a blockchain network to increase trust and trustworthiness.

The remainder of this paper is structured as follows: Section 2 provides a brief state of the art, highlighting the gap in the literature. Next, Section 3 presents the proposed system architecture and the method to include IoT devices. Then, Section 4 describes a case study validating our proposal, while in Section 5, we present and discuss the results of evaluating our proposed architecture with constrained devices. Finally, our conclusions are drawn in Section 6, highlighting possible future works.

## 2. Related Works

Mining activities that embrace the digital transformation will increase production, run more efficiently and effectively, and be more environmentally sustainable. In addition, they have the potential of setting new standards for workers’ health and safety and contribute to reskilling through educational and training programs. As an example, the Syama mine is a site in Mali that is benefitting from digitalization [7]. Resolute Mining took over operations at Syama in 2015, transforming it into the world’s first purpose-built automated mine. Employees use a fiber-optic network connected to aboveground control centers to manage and monitor all activities, from clearing the drill point to extraction, loading, and hauling. Another advance at Syama is Sandvik Automine for Trucks, officially released in December 2019, allowing the haul trucks to run autonomously underground using LiDAR and then switch to GPS when they reach the surface. Although the initial investment was steep, machines can now operate 22 h a day without time lost due to shift changes. Overall, the effects of digitization will cut mining costs by 30%, representing a true game-changer within the mining sector, especially for remote regions [8].

Currently, an increasing number of studies aim to cover the monitoring of machine or equipment parameters [9,10] with online and offline processing to detect or predict future failures, the definition of risk-assessment algorithms [11] to estimate the risk associated with different aspects to the mines (e.g., cyberattacks to mining equipment [12], workers’ safety [13], machine faults [14], etc.), operation monitoring [15], and so on. Along this line, Lööw et al. introduced the term Mining 4.0 [16] as the declination of the Industry 4.0 concept in the mining domain. Similarly, Chaowasakoo et al. [17] proposed and discussed a novel technique to plan the movement of shovels and trucks by adopting a digitized approach. Regarding the maintenance of assets, Carvalho et al. [10] used unmanned aerial vehicles (UAVs, i.e., drones) to inspect rollers in conveyor belts and detect possible failures using computer vision techniques.

In recent years, there has been a growing interest in integrating blockchain technologies into IT systems for enabling trustless architectures [18] in several application domains. Blockchain provides a trusted repository of information, where data are secure and traceable, and the data source can be precisely identified [6]. Thus, blockchain has the potential to enhance data security, traceability, accountability, integrity, transparency, and trustworthiness [19]. One of the first domains adopting blockchain-based systems with enthusiasm was insurance. According to authors of [20], blockchain could positively affect different processes, such as improving the customer experience and reducing operating costs. In this case, the cryptography primitives of blockchain could reduce the overhead related to manual data entry and verification. Despite the focus, these benefits also apply to several other application domains. For instance, authors of [19] explored the potential of blockchain in the automotive industry [19] for identity management and tamper-proof data management. Likewise, authors of [21] discussed the benefits of blockchain in the oil and gas industry for tracking, compliance, and data storage. Recently, authors of [22] discussed the use of blockchain technology to improve built asset sustainability through a comprehensive and detailed material traceability method. More related to our work, authors of [23] presented a blockchain-based system to store aircraft maintenance records, with a focus on security. Similarly, authors of [24] presented a blockchain-based framework for the maintenance of military equipment. Together, these studies provide important insights into the potential of blockchain networks to enable trust among unknown stakeholders by providing a transparent record of information in a decentralized way, removing the need for a trusted intermediary. Furthermore, the use of blockchain-based smart contracts makes asset monitoring and validation less human-dependent and prone to errors, while the inherent properties of blockchain increase the security and transparency of the transacted data.

Despite the growing interest in technology, integrating IoT and blockchain still faces several open challenges. As thoroughly described in [18], these challenges can be grouped into three major areas: privacy preservation, scalability, and utilizing blockchains in scenarios involving devices with constrained capabilities. For privacy preservation and scalability, the current research focuses on the trade-off between public and private blockchain networks or architectures using a combination of both [6]. In these scenarios, a certain level of trust among the system users exists, reducing some security concerns while increasing the overall performance.

Nonetheless, there has been little discussion about using blockchains with devices with constrained capabilities such as IoT sensors, which typically have stringent computational and networking limitations and a very restricted energy budget. On the one hand, IoT devices can be users of the blockchain, where blockchain provides support to IoT devices for access control, firmware updates, and other services in a decentralized way. In this case, the IoT does not impose demanding requirements, and the challenges are related to interfaces and implementation [18]. On the other hand, IoT devices can be a part of the blockchain data on-chaining system, as the reliable provisioning of blockchain-external data to smart contracts [25]. In this case, IoT devices are considered “hardware oracles” [26], meaning that they are the direct data sources of the physical phenomena they are sensing. Oracles have a tremendous responsibility to the blockchain-based systems as the insertion of incorrect information creates an immutable record that could trigger an irreversible action [20]. Therefore, these IoT devices face several challenges to become trustworthy oracles [25], such as reporting readings without sacrificing data security [19] while maintaining low-cost constrained computing capabilities [27].

In summary, these studies outline a clear need to understand the inherent challenges, issues, and limitations of integrating new technologies in an end-to-end mining inspection system. On the one hand, blockchain can provide a transparent and auditable repository of information, enabling trust between the unknown stakeholders involved in the inspection process. Nonetheless, current literature lacks a description of architectures to achieve this goal. On the other hand, IoT-based measuring instruments could work as trusted oracles that vouch for the truthfulness of the collected data during the inspection. However, there has been little quantitative analysis using low-cost IoT devices with blockchain-based systems, particularly for mining inspections.

## 3. Proposed System Architecture

We propose an architecture that integrates mobile devices, IoT sensors, and blockchain technology. The proposed architecture considers low-cost IoT sensing devices as direct actors on a blockchain network to guarantee a root of trust for the sensed data [25]. Furthermore, the integrity, auditability, and traceability of the sensed data are maintained and enforced by the blockchain network [28]. Here, we use blockchain technology as a decentralized trusted repository of information for several unknown and untrusted stakeholders, a proven use-case for blockchain [29]. Furthermore, we rely on existing blockchain implementations and protocols, as the type of consensus and the size of existing blockchains networks offer a more secure platform for developing new types of decentralized applications [30].

Due to practical restrictions, the proposed architecture has two main limitations. First, we only focus on measurements in the inspection process, as natural language processing for the observations is beyond the scope of this work. Second, even if the system considers several unknown stakeholders, we consider the OEM of tools and inspectors as trusted entities, and we focus on extending this trust to the other stakeholders in the system in a decentralized way.

**Figure 1:**
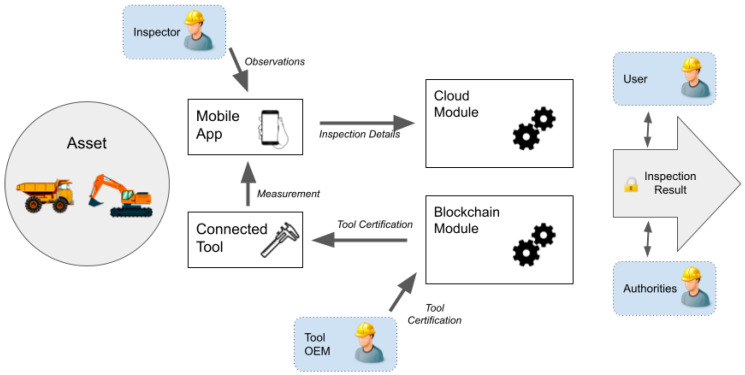
Proposed high-level architecture for a mining inspection system.

As shown in Figure 1, we consider an asset (e.g., truck, excavator) as the entry point of the information flow that ends with the production of an inspection report. The inspected asset is of interest to several actors (e.g., OEM of assets and tools, asset owners, users, on-field inspectors, authorities) that interact with the four main components of the architecture: the cloud module, mobile app, connected tool, and the blockchain module.

The cloud module supports the inspection business process (e.g., assets, inspections points, measurements, observations). It also holds the algorithms for evaluating safety-related risks and providing the inspection result. The mobile app benefits from portable devices (i.e., smartphones, tablets) and provides an interface to the onsite inspector to collect onsite measurements and observations. The mobile app automatically uploads the collected information to the cloud module when an Internet connection is available, minimizing the time overhead and possible errors due to manually writing the information. If no connection is available, the app can easily store the information of several inspections.

Our architecture considers measuring tools that precisely measure several physical parameters (e.g., dimensions, noise, heat, light) needed during on-field inspections. These devices are called connected tool and include new generation measuring instruments or legacy devices retrofitted with low-cost IoT platforms. The onsite inspector uses the connected tool that directly interacts with the mobile app using near-field communication capabilities, simplifying the process and reducing human intervention.

The last component is the blockchain module that gathers all the smart contracts representing assets, tools, inspections results, and certifications, providing a transparent record, auditable by all stakeholders. For blockchain operations, and aligned with current literature [26], the actors and devices in our systems are identified by their unique combination of public/private keys. More complex identity schemes are possible with blockchain, even realizing completely decentralized public key infrastructures [18]. However, this research topic is beyond the scope of this paper.

This blockchain identity makes several inspection actions accountable to a particular actor (e.g., inspector, operator). Likewise, the IoT device also has a blockchain identity in our architecture and generates digitally signed transactions. The use of cryptography at the root of the architecture addresses some of the current security challenges in IoT applications [31] and guarantees that information generated by the device reaches the blockchain unaltered. Furthermore, the blockchain identity of the device can be linked to the integrity of its hardware and firmware by using a physical unclonable function (PUF) [32], and the tool OEM can control firmware updates. Given that we consider the OEM of the tool as a trusted entity and the inspector as a trusted source, our architecture provides the missing component to convert a connected tool into a trustworthy oracle [27], ensuring data integrity by creating immutable, traceable, and non-repudiable records easily verifiable by other stakeholders. Moreover, the smart contracts on the blockchain module provide a decentralized, verifiable, and transparent way to manage several other elements of the inspection process, increasing their trustworthiness. For instance, certifications can be implemented as tokens, generated and managed by the certification authority, and directly linked to a particular operator or device. Another example is implementing a reputation score for the operators and inspectors, based on voting by previous users. Furthermore, the smart contract can automatize several steps of the inspection process. For example, the contract can implement simple logic, such as accepting a measurement for an inspection only if it is coming from a certificated tool or inspector, or more complex business rules, such as requiring more than one measurement from different tools or inspectors.

Our proposed system architecture makes noteworthy contributions to the current state of the art. First, it uses modern mobile devices (i.e., mobile app) to carry out onsite inspections and later automatically transfer them to the cloud module for further processing. Our proposed architecture can work even in remote mining sites with no Internet connection, as the current computing capabilities of modern mobile devices allow to store several hundreds of inspections. Second, it considers IoT-based measuring instruments (i.e., connected tool) as trusted oracles of a blockchain-based system (i.e., blockchain module). As a result, the connected tool becomes a practical choice to vouch for the truthfulness of the collected data during the inspection. Combining these technologies creates a cryptographically protected repository of inspection information, where data are immutable and traceable, and the data source can be precisely identified. This approach further improves trust and transparency between the stakeholders involved in the inspection process [3].

## 4. Use Case Validation

**Figure 2:**
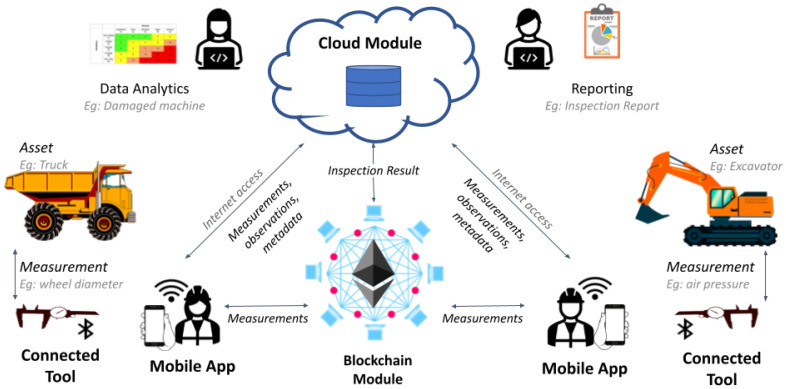
High-level architecture of the implemented use case.

In the context of an ongoing research and innovation project, we developed the proposed architecture as a multi-tier system, as shown in Figure 2. To this end, the architecture was tailored to fit the requirements of a real mining inspection scenario, allowing us to validate our proposal with the feedback of relevant companies in the mining sector. In the following paragraphs, we provide more implementation details of each component of the architecture.

### 4.1. Cloud Module

The cloud inspection platform is the core of the entire system. It hosts the machine registry (i.e., components of machines, vendors, machine models, etc.), inspector registry, inspection registry (i.e., inspection metadata, signed measurements, and observations) algorithms for the assessment of safety-related risks (e.g., a damaged machine may harm the worker that uses it), and an archive for the generated inspection reports (i.e., secure storage where PDF files of inspections are saved). This platform has been designed by following the micro-service methodology [33]; thus, the internal components interact among them and with the external entities, using a set of application programming interfaces (APIs). Moreover, a web-based user interface (UI) offers the possibility for users to insert, edit, and retrieve information about the machines and the inspection results.

### 4.2. Mobile App

The mobile app guides inspectors during the on-field operations, using a wizard-like navigation UI that allows selecting the machine to check, provide specific metadata (e.g., inspection date, machine-hours, next-inspection date, considered checkpoints), and record the measurements collected with the inspection toolkit and the observations about the asset status. Upon finishing the inspection, the mobile app allows reviewing the collected data before submitting it to the cloud module for storing and processing the inspection data. The mobile app requires network access (e.g., 3G/4G/5G, WiFi) to perform this operation; however, if no connection is available, the mobile app can locally store the data of several inspections.

The UI/UX of the mobile app has been designed by following the hierarchical data model of machines. First, each machine has multiple systems (e.g., engine); then, each system may have multiple subsystems (e.g., fuel subsystem); finally, each subsystem may have multiple checkpoints (e.g., fuel tank). On average, each machine has around 30–35 subsystems, and each subsystem has four checkpoints (deviation from 2 to 4) equally divided into measures and observations. Thus, an inspection contains, on average, between 140 to 160 checkpoints. Table 1 presents the checkpoint distribution per type of machine.

### 4.3. Blockchain Module

The blockchain module groups the smart contract needed to support our proposed system. Based on the “smart-twin” architecture proposed in [26], we developed two types of smart contracts: *Twin* and *Apps*. First, we used the *Twin* contract to represent the sensing device and the assets, keeping identification data (i.e., model, manufacturer, owner, and certifications). Then, we used the *App* contract to implement elements of the business logic, such as the result of the inspection and certifications. It is important to recall that these processes directly and automatically interact with the assets and the sensing tool (*Twin* contracts).

Each asset stores its measurements, which are unequivocally linked to a particular tool. The assets also store the inspection results linked to tools and inspectors. Certifications are represented with tokens, managed by the certifiers, and assigned to tools and assets. The algorithms to determine the outcome of inspections can be defined by the OEM or by local authorities. To implement this logic, we chose the Ethereum blockchain [34] because it is considered as the reference public blockchain implementation for smart contracts and can also function as a permissioned network. Nonetheless, migrating our implementation to a different blockchain platform with scripting capabilities should not be an issue. Finally, in our implementation, we adopted industry-approved libraries (i.e., OpenZepellin (https://github.com/OpenZeppelin/openzeppelin-contracts, accessed on 15 December 2021)) to reduce the security concerns deriving from the vulnerabilities that a smart contract may introduce in our system [35].

### 4.4. Connected Tool

The connected tool includes two types of devices capable of measuring different physic quantities: modern instruments (including wireless communication interfaces such as NFC or BLE) and legacy instruments (without wireless interfaces). One example of a modern instrument is the GARANT HCT IP67 caliper (https://www.hoffmann-group.com/p/412780, accessed on 15 December 2021), capable of storing digital readings and transferring them as comma-separated values using a Bluetooth interface. For the legacy instruments, we propose using a retro-fit kit based on low-cost IoT development platforms (e.g., Arduino, STM32, ESP32). As an example, we chose a low-cost RS digital caliper (https://it.rs-online.com/web/p/calibri/8412518, accessed on 15 December 2021), and we developed a “shield” that interacts with the tool, enabling a BLE communication interface. Compared to WiFi, BLE requires less energy, and even if NFC could provide even an easier user experience, NFC is not currently available on all mobile devices. To enable direct interaction of the connected tool with the blockchain system, we used our custom multi-platform library introduced in [26,27]. Furthermore, the retro-fit kit includes a small OLED screen to show the measurements to the user of the tool. Thus, the connected tool has three main layers: sensing (to interact with the legacy tool and display the value on the OLED), communications (to send the data to the mobile app using BLE), and blockchain (to perform the cryptographic functions required on blockchain networks). Finally, we opted for the Arduino IDE for our implementation as a developing platform, favoring cross-platform compatibility over code optimization.

## 5. Evaluation

To effectively evaluate our case study, we implemented it as a fully-working prototype. The cloud inspection platform was implemented using Docker (version 20.10.7-0ubuntu5 20.04.2) and Docker Compose (version 1.29.2) as containerization engine and multi-container manager, respectively. The platform was deployed on a cloud virtual machine (Digital Ocean General Purpose droplet) based on Ubuntu 20.04.3 LTS with 8 GB of RAM, 160 GB of SSD, and 4vCPU.

Then, the blockchain module runs on nodes using the official Geth client (version 1.10.1-stable) on independent virtual machines with 4 GB of RAM, 20 GB of SSD, and 4 vCPU on an OpenStack server using a clean Linux Ubuntu installation (version 18.04).

For the IoT platform, and based on the results and evaluation presented in our previous works [26,36], we opted for an ESP32 microcontroller. In particular, we selected the WRover-E 32-Bit ESP32 as the most suitable microcontroller in the 10 USD cost range. The board has an 80 MHz clock chipset with 1024 KB of program space and 320 KB of memory. It also includes Serial, USB, Wifi, and BLE interfaces for communication.

Finally, the mobile app was installed and tested on mobile devices running the Android OS version 9.

### 5.1. Inspection Report Footprint

Mining operations typically occur in remote locations, where network access might be limited and expensive. Therefore, estimating the traffic needed at the edge of the mining application is important. To this end, we evaluated the average data needed to upload an inspection to the cloud platform. The number of measurements and observations collected during an inspection depends on the actual status of the inspected machine and its checkpoints, as shown in Table 1. To preserve the confidentiality of information collected during the pilot, we statistically estimated the size of reports using a Monte Carlo approach.

First, we modeled the number of checkpoints per subsystem of each machine type as a random variable with *truncated Gaussian* distribution by starting from a *Gaussian* distribution with mean and standard deviation defined in Table 1. Then, we modeled the aging, and, consequently, the lifetime of a generic machine, as an integer number $N_{age}$ between 1 and 100: 1 means that the machine is new, while 100 means that the machine is at the end of its useful life. Next, we carried out 100 different campaigns of report generation by choosing 100 different random seeds (i.e., seeds computed from timestamp) to not stick to one particular “lucky” seed. For each campaign, we generated 250 reports as follows: first, we randomly chose one of the 12 machine types (Table 1), then we sampled from a uniform distribution between 1 and 100 the normalized age $N_{age}$ of the machine. Following that, we sampled, for each of the subsystems of the target machine, an integer number from the distribution of checkpoints as the number of checkpoints to be inspected. Then, for each checkpoint, we extracted a random integer *N* between 1 and 100. Assuming that, for each checkpoint, we can collect only one measurement or one observation, if $N<N_{age}$, we assumed that we collected an observation associated with the checkpoint; otherwise if $N>=N_{age}$, we collected a measurement. In this way, it is more likely to have more observations than measurements for an old machine.

At this point, we have a population of 25,000 inspections reports and we can statistically characterize the amount of KB required to transmit them to the cloud. Inside the mobile app, the inspection data has the following structure:

- Each measurement is represented as a *double* variable (8 bytes) for the actual value and an *integer* variable (4 bytes) for the ID of the unit of measure.
- Each observation is represented as a *string* with length 255 chars (1 char is equal to 1 byte).
- Each checkpoint is represented as a *integer* variable (4 bytes) that contains the ID.
- The report contains also the ID of the inspector (*integer*, 4 bytes), the machine ID (*integer*, 4 bytes), the machine working hours (*integer*, 4 bytes), the inspection timestamp since the Unix Epoch (*integer*, 4 bytes), and the timestamp of the next inspection (*integer*, 4 bytes).

**Figure 3:**
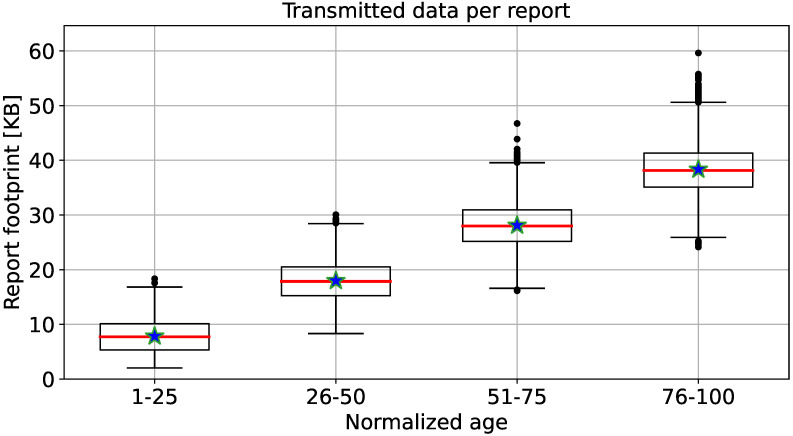
Box-plot of the report footprint (from mobile app to cloud module) with respect to the aggregated normalized age. Median values are red lines, mean values are blue stars, and outliers are black dots.

Figure 3 shows the box plots of reports size in KB against the normalized age of the target machines. There, for the sake of visualization, we split the sample distribution into four groups, according to the normalized age of the machines. Therefore, normalized ages between 1–25 could represent new machines; fairly new and fairly old machines are represented by normalized ages between 26–50 and 51–75, respectively; normalized ages between 76–100 could represent old machines. Then, for each sample distribution, the upper and lower quartiles are represented with a box, and the whiskers represent the lowest and highest values of the report footprint distribution. From the data, we can highlight that when the machine is getting older, the size of the report increases. This is due to how the information about the machine status is reported. If the machine is “young”, we collect more numerical readings (i.e., double numbers or integers with 8- or 4-bytes length, respectively) than observations (i.e., text with a prefixed length of 255 bytes). When the machine is getting old, we collect more observations than numerical readings. This turns into an increasing size of the report proportional with the aging. More details about statistics of the report sizes are available in Table 2.

An Internet connection is needed to send the report from the mobile app to the cloud module for processing and persistence. Thus, sending a report is not a concern when traditional Internet access such as cellular networks (over edge, 3G, 4G, or 5G) or broadband access (over WiFi) is available. However, as mines are usually located in remote locations, a cellular/fixed Internet connection may not be available. For this reason, we evaluate using satellite communication equipment to send data to the cloud. Usually, this type of communication requires a data plan billed per MB of data. Assuming that one MB of data sent via satellite communication costs USD 6.80 (https://www.bluecosmo.com/inmarsat-bgan-monthly-service-plan.html, accessed on 15 December 2021). Table 3 shows the estimated costs of sending one inspection report using this communication technology to the cloud, net of overheads such as IP, TCP, TLS, and HTTP headers.

### 5.2. Connected Tool Footprint

**Figure 4:**
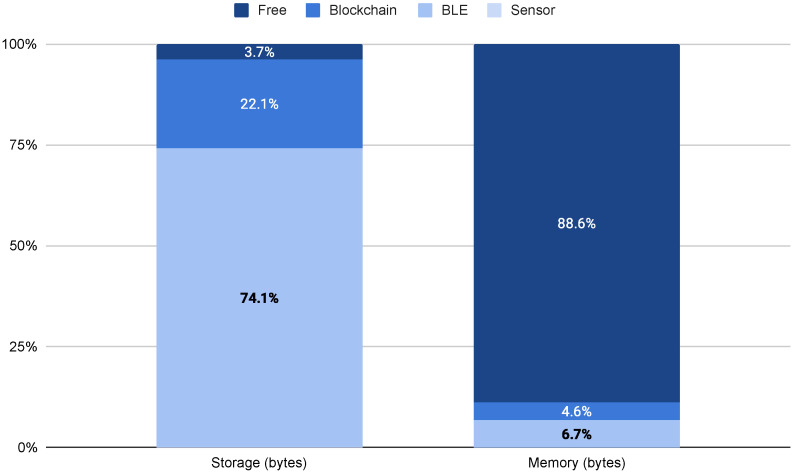
Disk and memory usage normalized to the total available.

Based on the statistics provided by the compiler and incrementally adding the functions required by the connected tool (i.e., sensor, BLE, blockchain), the results in terms of disk and memory usage are shown in Table 4 and Figure 4. There, absolute values are expressed in bytes, while normalization is performed against the total available disk and memory. From Figure 4, we observe that the heaviest footprint is for disk usage of the BLE, with almost 75%, while Blockchain operations account for less than 23%. Nonetheless, there is almost 5% of free space in the device, while the total memory usage is less than 12%. Moreover, it is important to recall that using the Arduino IDE adds approximately 15% of resources overhead. We can conclude that the selected IoT board provides a suitable, cost-effective platform for developing integration kits for measuring tools with legacy data interfaces without BLE, NFC, or WiFi.

### 5.3. Transaction Costs

Using the information provided by *Geth*, we obtained the amount of gas needed for creating the two types of contract (i.e., *Twin* and *App*) and the different transactions needed. On public Ethereum networks, this gas cost translates into monetary cost by setting a gas price in Ethereum cryptocurrency (ETH) and using the current exchange value of ETH. Similar to [26], we considered the gas price of 10 *gwei*. The exchange of cryptocurrency is quite volatile, and its accurate estimation goes beyond the scope of this paper. However, historic values can provide a good reference for evaluating different scenarios. As an example, we consider the average yearly exchange price reported by Etherscan (https://etherscan.io/chart/etherprice, accessed on 15 December 2021) for 2019, 2020, and 2021. Thus, Table 5 shows the amount of gas for the transactions and the monetary costs in USD, using three different exchange rates for USD/ETH: USD 182 for 2019, USD 307 for 2020, and USD 2778 for 2021.

The results show that the price volatility drastically changes the costs of the system. However, the most common operation (measurement) is less than one dollar, even with the highest exchange rate. Moreover, an immutable inspection report will cost less than USD 5 in the worst case.

### 5.4. Transaction Processing Time

Using the gas price of 10 *gwei*, we deployed a tool contract in an Ethereum live test network (Ropsten) and evaluated the real transaction processing time for registering a measurement. This transaction, created by the connected tool, has an average size of 136 bytes and a gas cost of 27,300. We focused on testing this operation because it is the most frequent transaction in the architecture. We sent 200 transactions, approximately once per hour for one week.

**Figure 5:**
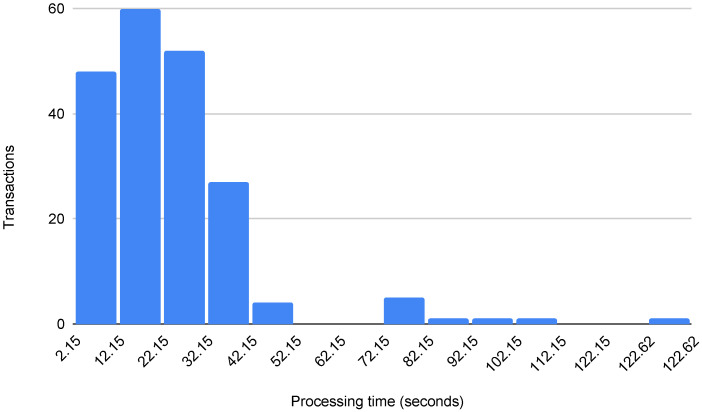
Distribution of the processing times on a public blockchain (expressed in seconds).

Figure 5 shows the distribution of the processing times of the 200 transactions. The average blockchain processing time was 24 s, with a median of 21 s. These delays are within the fastest processing times currently possible on public blockchain networks. Moreover, in this experiment, only three transactions took more than 90 s to be processed (i.e., less than 2% of the total). It is important to notice that, typically, there is no monetary cost associated with a transaction on a private blockchain network. However, even if a private blockchain provides auditability and offers better performance (e.g., lower latency, higher transaction throughput), it is not entirely decentralized or as censorship-resistant as a public blockchain [37].

### 5.5. Power Consumption and Energy Requirements

Using an Otii device (https://www.qoitech.com/, accessed on 14 December 2021) we could measure the power consumption with an accuracy of ± (1% + 0.5 μA) at 3.3 V, at a rate of 1000 samples per second. Then, we consider 30 s as the time needed for an inspector to read the measurement on the device. We measured three cases: (i) baseline, where the measurement is only shown on the OLED display (i.e., sensing), (ii) BLE, where the measurement is sent to the app using BLE (i.e., communications), and (iii) Blockchain, where the device digitally signs the measurement before sending it to the app (i.e., Blockchain). Table 6 shows the minimum, maximum, average power consumption (in mA), and total energy consumption (in mWh) as the average of 100 experiments for each case. It is important to note that no low-power consumption optimization was implemented on the device. This data shows that enabling BLE increases the average power requirement by almost 38% (from 40.5 to 55.7 mA) and the overall energy consumption by 34%. Compared to BLE, enabling blockchain technology has almost no influence on power and energy requirements.

In summary, these results show that the device could work continuously for 3.72 h in the baseline case, 2.76 h when using BLE, and 2.75 h when adding blockchain functionality, with a 500 mAh rechargeable LiPo battery. In the context of a real mining inspection, and based on the information described in Section 4.2, an inspection should perform between 60 and 70 measurements. Thus, if each measurement is 30 s, the total operation time of the connected tool, using BLE and blockchain, will be only between 30 to 35 min.

## 6. Conclusions and Future Works

This paper presented and evaluated an end-to-end system to conduct mining inspections. The proposed system architecture uses off-the-shelf mobile devices and integrates IoT and blockchain technologies. Furthermore, our proposal provides a method to create smart measuring tools using low-cost embedded CPUs that directly interact with a blockchain system, guarantee data integrity, and increase trustworthiness. We highlighted the benefits of the proposed architecture by describing and evaluating the implementation of a pilot in a real mining inspection scenario. Such a pilot represents one of the outcomes of an ongoing research and innovation project funded by the EU Commission and involving research institutions, universities, and relevant companies in the mining sector. Our results showed that a very cost-effective IoT board (USD 10) could provide a suitable platform (in terms of disk usage and memory) to create new types of connected tools that directly benefit from blockchain technology. Furthermore, in terms of additional processing time, the operations performed on the blockchain averaged 24 s on a public blockchain network. Finally, concerning energy requirements, the connectivity of the tool (i.e., BLE) increased the average power requirement by almost 38%, while enabling blockchain had very little influence. Nonetheless, in the context of a full mining inspection and using a small 500 mAh battery, the connected device consumed only 20% of the total available energy. These findings suggest an important role for portable mobile devices, connected measuring tools, and a blockchain infrastructure in promoting the adoption of digital technologies to make inspection procedures more automated, reliable, and simple.

Future works include evaluating other communications methods between the connected tool and the mobile app (i.e., NFC, WiFi). Another interesting research direction is evaluating low-power modes and other IoT hardware platforms to reduce the power consumption of the connected tool. Finally, another interesting aspect to further develop the system is the integration of more complex evaluation algorithms (i.e., AI-based) that can run outside the blockchain. These algorithms could benefit from the information collected both on the cloud module and the blockchain module and should maintain the same level of trustworthiness.

## Figures and Tables

**Table 1:** Number of measures/observations that need to be collected per subsystems (assumption: one measure/observation per checkpoint).

Machine Category	Subsystems Per Machine	Min/Max Checkpoints Per Subsystem	Average Checkpoints Per Subsystem	Deviation of Checkpoints Per Subsystem
Articulated mining truck	38	1/9	4.13	2.21
Backhoe loader	34	1/13	4.71	3.03
Bolting rigs	37	1/9	4.11	2.46
Dozer	30	1/18	4.47	3.77
Hydraulic power shovel excavator—back hoe	31	1/12	4.16	3.00
Hydraulic power shovel excavator—front hoe	34	1/15	4.47	2.98
LHD	34	1/15	4.12	2.89
Rigid mining truck	38	1/8	4.11	2.12
Tracked drilling rig	35	1/12	4.11	2.67
Tracked loader	30	1/20	4.47	3.88
Wheel loader	34	1/12	4.50	2.69
Wheeled drilling rig	37	1/9	4.11	2.46

**Table 2:** Report footprint (expressed in KB) statistics with respect to normalized age (pure number).

Normalized Age	Mean	Std Deviation	Median	Max	Min
1–25	7.83	3.04	7.72	18.34	2.02
26–50	17.97	3.59	17.85	30.05	8.32
51–75	28.10	4.12	28.00	46.73	16.15
76–100	38.33	4.66	38.14	59.62	24.15

**Table 3:** Estimated cost (expressed in USD) of sending reports to the cloud using a satellite communication.

Normalized Age	Mean	Std Deviation	Median	Max	Min
1–25	0.05	0.02	0.05	0.12	0.01
26–50	0.12	0.02	0.12	0.20	0.06
51–75	0.19	0.03	0.19	0.32	0.11
76–100	0.26	0.03	0.26	0.41	0.16

**Table 4:** Footprint for the device module (expressed in bytes).

	Available	Sensor	BLE	Blockchain
Disk	1,310,720	1612	971,369	289,318
Memory	327,680	176	22,108	14,996

**Table 5:** Estimated transaction costs (expressed in USD) using different ETH/USD based on historical prices.

Transaction	Gas	ETH/USD 2019	ETH/USD 2020	ETH/USD 2021
Twin (Tool)	200,412	0.36	0.62	5.57
Measurement	27,300	0.05	0.08	0.76
App (Inspection)	3,500,531	6.37	10.75	97.24
Inspection Report	157,800	0.29	0.48	4.38

**Table 6:** Power requirements and energy consumption (at 3.3 V) in 30 s windows for three cases.

		Min. (mA)	Max. (mA)	Avg. (mA)	Energy (mWh)
(1)	Baseline	40.2	41.6	40.5	134.4
(2)	BLE	42.2	154.1	55.7	181.2
(3)	Blockchain	42.3	154.2	55.8	182.4

## Data Availability

Not applicable.
